# Development and validation of the Interoceptive States Static Images (ISSI) database

**DOI:** 10.3758/s13428-021-01706-2

**Published:** 2021-10-14

**Authors:** Federica Biotti, Sarah Ahmad, Racquel Quinn, Rebecca Brewer

**Affiliations:** Department of Psychology, Royal Holloway, University of London, Egham, Surrey TW20 0EX UK

**Keywords:** Interoception, Internal states, Bodily signals, Static images, Social interaction

## Abstract

Internal bodily signals provide an essential function for human survival. Accurate recognition of such signals in the self, known as interoception, supports the maintenance of homeostasis, and is closely related to emotional processing, learning and decision-making, and mental health. While numerous studies have investigated interoception in the self, the recognition of these states in others has not been examined despite its crucial importance for successful social relationships. This paper presents the development and validation of the Interoceptive States Static Images (ISSI), introducing a validated database of 423 visual stimuli for the study of non-affective internal state recognition in others, freely available to other researchers. Actors were photographed expressing various exemplars of both interoceptive states and control actions. The images went through a two-stage validation procedure, the first involving free-labelling and the second using multiple choice labelling and quality rating scales. Five scores were calculated for each stimulus, providing information about the quality and specificity of the depiction, as well as the extent to which labels matched the intended state/action. Results demonstrated that control action stimuli were more recognisable than internal state stimuli. Inter-category variability was found for the internal states, with some states being more recognisable than others. Recommendations for the utilisation of ISSI stimuli are discussed. The stimulus set is freely available to researchers, alongside data concerning recognisability.

## Introduction

Internal bodily signals, such as hunger, thirst, fatigue, nausea, pain, temperature, and cardiac and respiratory signals, are essential for human survival, indicating the physiological state and functioning of the body (e.g. the sensation of thirst signalling the level of dehydration in the body). The ability to perceive and identify these internal sensations, known as interoception (Craig, 2003a), is fundamental to multiple psychological processes, such as emotional processing (e.g., Critchley & Garfinkel, 2017; Garfinkel & Critchley, 2013; Schachter & Singer, 1962; Seth, 2013), and learning and decision-making (Bechara & Damasio, 2002; Dunn et al., 2010; Werner et al., 2009). Furthermore, a growing body of research has linked interoception to mental health and subjective wellbeing; atypical perception of interoceptive states has been found in several mental health conditions and neurodevelopmental disorders, such as Eating Disorders (Klabunde et al., 2013; Pollatos et al., 2008), autism (Garfinkel et al., 2016; Hatfield et al., 2019; Mul et al., 2018; Nicholson et al., 2019), anxiety and Panic Disorder (Ehlers, 1993; Paulus & Stein, 2006; see Khalsa et al., 2018 for a review), depression (Dunn et al., 2007; Furman et al., 2013; Forrest et al., 2015; Harshaw, 2015; see Eggart et al., 2019 for a review), and schizophrenia (Ardizzi et al., 2016). Given the vital role of interoception in understanding typical emotion processing and learning and decision-making, as well as its atypicality in several mental health conditions, research on interoception and emotion has grown significantly in recent years.

While numerous studies have focused on the perception of interoceptive states in the self, very few (e.g., Kaulard et al., 2012) have researched the recognition of these states in others, beyond the domain of affective emotion (e.g., happiness, anger, sadness). Recognition of others’ affective emotional states (which feature an interoceptive component; Schachter & Singer, 1962) has been studied in detail, in typical adulthood, clinical samples, and across development; indeed a PubMed search using the term “emotion recognition” generated 15,009 results. Recognition of others’ emotional states is crucial for successful social interactions, as well as building and maintaining relationships, making it an important area for psychological research. Recognition of interoceptive states (beyond the affective domain) in others, including identifying others’ hunger, nausea, pain, and breathlessness, for example, is presumably equally important for social interaction, and arguably more important from an evolutionary perspective (as identifying perturbations in these states is necessary in order to offer care and assistance to others). Similarly, studying the mechanisms behind the ability to recognise other people’s bodily sensations is crucial to improve our understanding of empathy for these states in others, with important theoretical and clinical implications. It is somewhat surprising, therefore, that research has, thus far, neglected to investigate this ability.

One reason for the dearth of research investigating the recognition of others’ non-affective internal states is presumably the lack of available stimuli. Compared to affective emotion recognition research, the lack of stimuli for the investigation of non-affective state recognition is striking. Since the publication of the “Pictures of Facial Affect” (Ekman & Friesen, 1976), the first standardised battery of facial emotion stimuli, several databases of visual stimuli depicting facial and bodily affective expressions have been developed (e.g., Beaupré et al., 2000; Langner et al., 2010; Lundqvist et al., 1998; Matsumoto & Ekman, 1988; Volkova et al., 2014; Wingenbach et al., 2016). Visual stimuli depicting facial and bodily expressions of affective states have been a key component of emotion research, and substantially contributed to our knowledge of affective and cognitive neuroscience, and social and clinical psychology. A purpose-built battery of stimuli depicting non-affective interoceptive states in others will enable research on social cognition to investigate the ability to perceive and recognise these signals in others. This will lead to an expansion of our theoretical understanding of the constructs of interoception and social perception in typical adult populations, developmental samples and clinical groups, both at the behavioural and neurological levels.

This report presents the development and validation of the Interoceptive States Static Images (ISSI), a database of full body static images of actors expressing either a non-affective interoceptive state or a control action, which is freely available to other researchers. The battery consists of 423 stimuli, which depict eight actors expressing various exemplars of nine internal states and nine control actions. All photos were taken from a frontal view in a controlled environment, and underwent a standardised image processing procedure to control for lighting conditions, size, position, and background. Stimuli were validated in two stages, one utilising free labelling and the other utilising visual analogue rating scales. Recognition data for each individual stimulus and for the state and control actions overall, including the extent to which they are confused with each other, are provided.

## Methods

### Stimulus development

#### Actors

Eight trained actors (four female) aged 22 to 48 were recruited through online and campus advertisement. Neither ethnicity nor first language was specified as a recruitment criterion, but they were recorded. No specific ethnic group was targeted for recruitment and no actors were excluded based on their ethnicity. The recruitment was interrupted once the necessary number of actors was reached. All the actors who responded to our recruitment call reported being of Caucasian ethnicity. Actors were either drama students or had previously completed acting training. Actors were informed about the procedure and the purpose of the stimulus set, and gave their consent to take part in the recording session and for their images to be used in scientific research, presented at conferences, published in academic journal articles, and shared with other researchers. Actors received financial remuneration for their time.

#### Procedure

Prior to the recording session, actors were provided with the list of the internal states and actions they would be required to perform, and were asked to practice depicting each state or action prior to the recording session. During the recording session, they were required to wear black trousers, black socks, and a black t-shirt. Female actors were asked not to wear make-up and to tie their hair to ensure their face was completely visible at all times. Photos were taken in a purpose-built photography studio. Actors stood in a specified position in the centre of a white background, facing a camera placed on a tripod. Softbox LED lighting was used to control lighting conditions across different shooting sessions, and to reduce shadows.

Actors first produced ten control actions (jumping, clapping, lifting, running, washing hands, spinning/twirling, stumbling, walking, waving, beckoning) and then expressed ten non-emotional internal states (cold, fatigue, nausea, pain, breathlessness, hunger, thirst, hot, satiety, itch). For each stimulus category, the actor was asked to practice before posing the state or control action five separate times, which were used as different exemplars of the same stimulus. Between each attempt, the actor was asked to re-set to a neutral body position and to re-position in the middle of the background. Between each stimulus type, a longer break was given to allow actors to rest and prepare for the next stimulus category. The order of stimulus production was fixed and did not vary across actors.

#### Image processing

Raw photos were edited in Adobe Photoshop 2019. The backdrop was replaced with an artificial white matte background. Image artefacts and distracting visual information (e.g. tattoos) were removed. Brightness and contrast were adjusted and standardised across different images. Sharpness was increased with the function Smart Sharpness by 309%, with 0.6-pixel radius, 100% noise reduction, and by removing lens blur.

Image size and actor position were matched across stimuli using a 3456 × 5184 pixels white template. The first stimulus was positioned in the centre of the template to reach the desired size, which was sized to subtend 12° of visual angle vertically when viewed at 60 cm. Guidelines were drawn to delimit the boundaries of the actor in this position (extremes of head and feet in the vertical axis and extremes of right and left shoulders in the horizontal axis) and to provide a frame of reference for all the subsequent stimuli. For each actor, images were layered onto the original template and the size and position of the actor was adjusted to fit these guidelines. Each layered image was saved as a new file.

### Stimuli validation

All stimuli went through a pre-selection process based on basic visual properties by the researchers. Photos where parts of the actor’s body were missing (e.g. the head being outside the picture top edge in some ‘jumping’ exemplars), or where motion blur could not be resolved through editing, were removed from the database. This resulted in all stimuli depicting the control action ‘stumbling’ being removed, due to a high proportion of images including several of these issues. To retain an equal number of control actions and internal states, stimuli depicting ‘thirst’ were also removed, owing to actors reporting that this state was difficult to portray because of the lack of a visible behavioural response to feeling thirst, and the authors agreeing that stimuli were not recognisable as depicting thirst. For each actor, and for each stimulus category, the four exemplars with the highest visual quality, judged by the researchers, were selected to be included in the first validation task, yielding a total of 560 stimuli.

#### Stimulus selection: Free-labelling task

Forty participants (four male) aged 18–30 years (*M* = 19.05, *SD* = 2.68) were recruited through Royal Holloway, University of London (RHUL) SONA System to take part in a free-rating task. Participants were all students at RHUL and received course credits for their participation. There were no exclusion criteria for this task, although any diagnosis of a mental health condition was recorded. A general description of the task procedure was provided but participants were not informed of the aim of the study until the end of the session, to avoid influencing their responses. Stimuli were divided into two sets of 280 images. Participants viewed one of the two sets, in order to reduce fatigue. Instructions were standardised across participants and the experimenter provided them verbatim as follows: *You will see a series of body postures, one by one. For each one, you need to provide a very brief description of what you think the body posture represents (for example what the person is doing, thinking or feeling). There will be many stimuli, so it’s very important that you keep your answers as brief as possible. Ideally, you will use a single word or a short phrase. For example, if you see an image depicting a person sneezing, you can simply answer ‘sneezing’. If you think that the person could be doing, thinking or feeling more than one thing, you can give multiple answers, but please try to keep the description of each one brief. If I need more details, I will ask for them. There are not right or wrong answers, so I will not provide any feedback during or after the session. I will simply record your answers and occasionally intervene if I think something is not clear or if I need more details*. Following the instructions, participants were invited to ask any questions they may have about the procedure. Then, the experimenter sat a few meters behind the participant and typed their responses verbatim. When additional information was required, the experimenter used standardised phrases to prompt the participant. If the answer required more details, the experimenter would say “*Can you tell me more about that?*”. If the answer was ambiguous/unclear, the experimenter would say “*Can you be more specific?*” or “*Can you tell me what you mean by that?*”. Finally, if participants’ responses were too verbose, the experimenter would say “*Try to use single words or short phrases*”. This task took approximately 20 min to complete.

#### Stimulus validation: Label selection and rating task

**Fig. 1:**
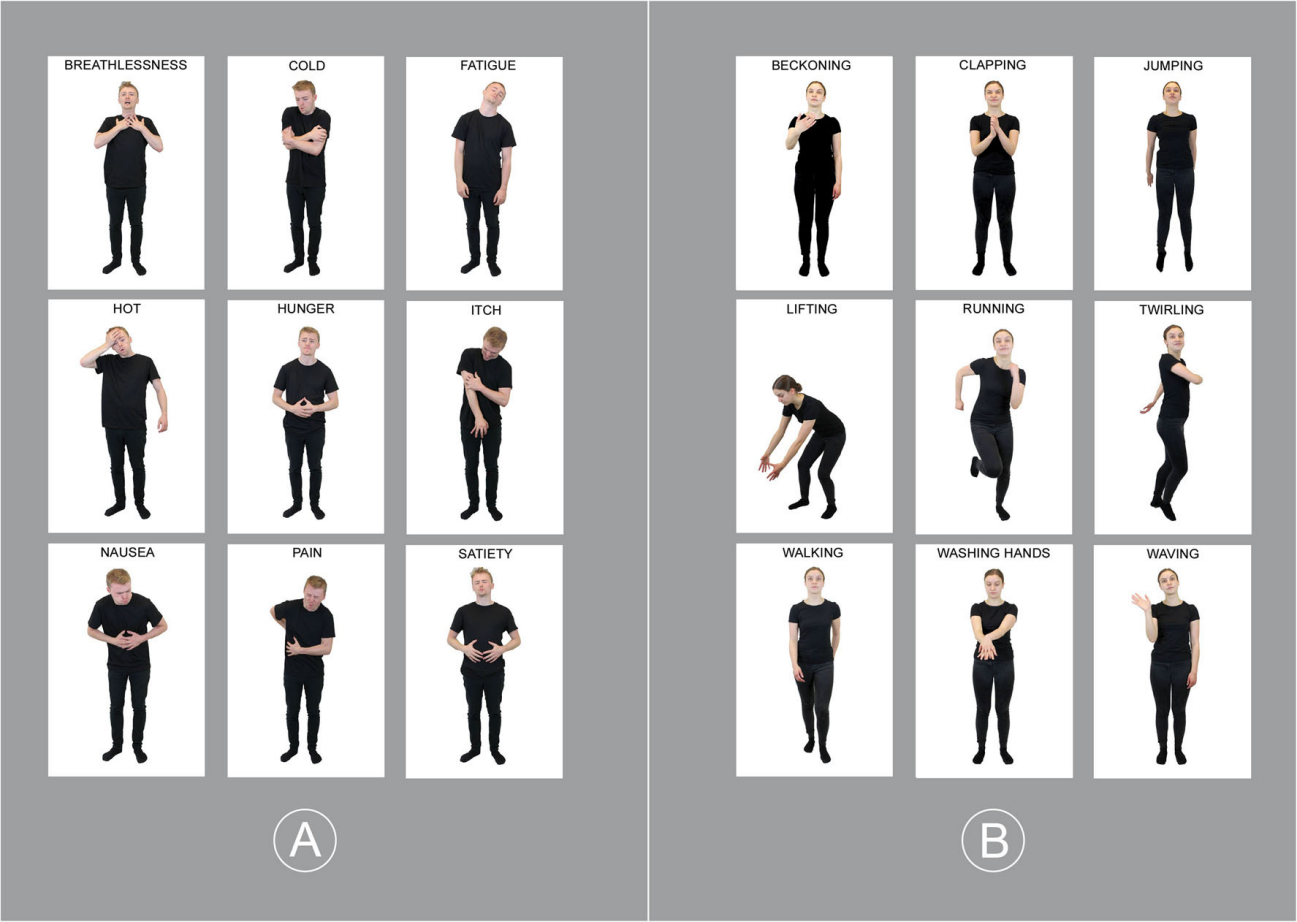
Examples of stimuli from the ISSI database. **a** Examples of internal state stimuli. **b** Examples of control action stimuli

Based on the results of the free-rating task, 423 stimuli were selected to be used in the second step of validation (details on the selection procedure can be found in the Results section). Of these, 202 stimuli depicted nine internal states (breathlessness, cold, fatigue, hot, hunger, itch, nausea, pain, satiety) (Fig. 1a) and 221 stimuli depicted nine control actions (beckoning, clapping, jumping, lifting, running, twirling, walking, washing hands, waving) (Fig. 1b). Participants were recruited from the RHUL Sona System, Testable Minds database (www.testable.org), and through advertisements on social media. A total of 412 participants (169 female) aged 18–71 years (*M* = 30.08, *SD* = 10.56) with no diagnosis of any mental health condition took part in an online labelling task. The task was programmed in Testable and presented participants with a random sample of 100 stimuli. On each trial, a single image was presented in the centre of the computer screen and remained visible until participants had finished responding. Participants were provided with a list of the nine internal state and nine action labels (presented in alphabetical order) and asked to select which label best described the image. If they were unsure, participants could select more than one label or skip to the next trial if they thought that no label applied. Following label selection, participants were prompted to rate how well each chosen label described the image, using a five-point Likert scale (*Very Poorly; Poorly; Moderately; Well; Very Well*). This task took approximately 30 min to complete.

## Results

### Free-labelling task

Participants’ responses in this stage were analysed qualitatively. First, two coders independently coded the responses for accuracy (referring to identification of the intended state or action). A score of 1 was given to those responses which either correctly identified the state or action or, for state stimuli, correctly described the action portrayed, associated with the state (e.g. for the ‘fatigue’ stimuli, both ‘tired’ and ‘yawning’ were considered correct responses). A score of 0 was given to inaccurate responses (e.g. ‘hot’ or ‘shocked’ to describe a ‘breathlessness’ stimulus). In instances where coders disagreed, responses were discussed by all authors until an agreement was found. Inter-coder agreement was near perfect (*k* = 0.81).

Each stimulus was given a *recognisability index*(RI) which corresponded to the mean accuracy score. Overall, internal state and action stimuli were recognised correctly 65% and 75% of the time, respectively. Of the internal states, itch (*M* = 88%, *SD* = 13%, range 55–100%) and cold (*M* = 88%, *SD* = 15%, range 55–100%) were the best recognised states, while hunger was the least well recognised state (*M* = 22%, *SD* = 8%, range 10–40%). Among the control actions stimulus set, walking was the best recognised state (*M* = 91%, *SD* = 12%, range 55–100%), whereas beckoning stimuli were the least well recognised (*M* = 49%, *SD* = 11%, range 30–75%). See Table 1 for a full summary of RIs.

**Table 1:** Recognisability Indices (RI) for each Internal State and Action category. RIs represent the proportion of recognition accuracy in the free-labelling task (Stage 1)

	%RI mean (SD)	%RI minimum	%RI maximum
BREATHLESSNESS	29 (16)	5	65
COLD	88 (15)	55	100
FATIGUE	80 (18)	45	100
HOT	35 (19)	5	65
HUNGER	22 (8)	10	40
ITCH	88 (13)	55	100
NAUSEA	70 (15)	40	95
PAIN	79 (20)	30	100
SATIETY	39 (16)	15	70
BECKONING	49 (11)	30	75
CLAPPING	85 (15)	55	100
JUMPING	78 (14)	50	100
LIFTING	65 (23)	10	100
RUNNING	84 (15)	55	100
TWIRLING	66 (16)	45	95
WALKING	91 (12)	55	100
WASHING HANDS	57 (10)	30	75
WAVING	80 (12)	60	100

Based on the RI, each stimulus was categorised according to recognisability, into five categories: *Very poor* (RI scores 0.0–0.2), *Poor* (RI scores 0.21–0.4), *Average* (RI scores 0.41–0.6), *Good* (RI scores 0.61–0.8), and *Very good* (RI scores 0.81–1). All stimuli categorised as *Very good*, *Good*, and *Average* were kept in the final database. In addition, we retained a minimum of two exemplars per actor for each stimulus category. For stimulus categories where fewer than two stimuli for a given actor were categorised as *Very good*, *Good*, or *Average*, the two stimuli with the highest RI were retained. See Appendix Table 3 for RI scores for every retained stimulus. A final set of 423 stimuli was retained and used in the second stage of validation^1^. In this final stimulus set, 209 stimuli depicted male actors, whilst 214 depicted female actors. Each of the eight actors was present in at least 50 stimuli, and the most depicted actor appeared in 56 stimuli.

### Label selection and rating task

#### Quality and Accuracy Scores

Each stimulus was rated by a mean of 97 participants (Min = 74, Max = 123). There are multiple ways in which the validity and quality of stimuli can be defined, so to allow researchers to select stimuli based on their own requirements, a comprehensive range of stimulus measures has been created and is provided below. For each stimulus, five separate scores were calculated: the *quality index*(QI); the *specificity index*(SI); the *maximum-distractor specificity index* (SI+), the *choice rate*(CR); and the *high-quality choice rate* (CR+) (Table 2). The scores were calculated based on the ratings of the whole sample (both female and male observers), as well as on ratings of female and male observers separately.

**Table 2:** Summary of scores. *T* = target; *D* = distractor. In the formulae for QI, SI, and SI+ *T* and *D* correspond to a value between 0 and 5 (participants’ ratings of how well a stimulus depicts a given state label). In the formulae for CR and CR+, *T* and *D* correspond to a binary value: 0 or 1 (indicating whether the label was selected (1) or not (0)). *n* = total number of stimulus ratings across all participants. *i* = ‘for all individual stimulus ratings across all participants’

Score	Abbreviation	Description	Formula	Range	Interpretation
Quality Index	QI	How well the target label describes the image	$\frac{\Sigma\ Ti}{n_i}$	0 – 5	0 = target label not selected 1 = very poor depiction 5 = very good depiction
Specificity Index	SI	How well the target label describes the image, over and above distractor state/action labels	$\frac{\Sigma \left({T}_i-\left(\frac{\Sigma\ Di}{n}\right)\right)}{n}$	-5 – 5	Negative values: target label received a *lower* rating than distractor labels taken together 0 = target and distractor labels are rated equally Positive values: target label received a *higher* rating than distractor labels taken together
Maximum-distractor Specificity Index	SI+	How well the target label describes the image, over and above the distractor receiving the highest rating	$\frac{\Sigma \left({T}_i-{D_i}_{max}\right)}{n}$	-5 – 5	Negative values: target received *lower*rating than distractors with highest rating 0 = target and distractors are rated equally Positive values: target received *higher*rating than distractors with highest rating
Choice Rate	CR	Proportion of raters who selected the target label, regardless of the quality rating	$\frac{{{\Sigma T}_i}_{selected}}{n}\times 100$	0% – 100%	0% = target label was never selected to describe the stimulus 100% = target label was always selected to describe the stimulus
High-quality Choice Rate	CR+	Proportion of raters who gave the target label (rather than a distractor label) the highest quality rating on that trial	$\frac{{{\Sigma T}_i}_{max}}{n}\times 100$	0% – 100%	0% = target label was never rated higher than distractors when describing the stimulus 100% = target label was always rated higher than distractors when describing the stimulus

The QI is a score ranging between 0 and 5, and was computed by taking the mean (across all stimulus ratings) of all quality judgements given to the target (intended) label. A score of 0 was assigned whenever the target label was not selected. The QI therefore reflects the extent to which the target label is perceived as describing the image well. High QI scores indicate that the target label describes the image very well. Conversely, lower QI scores indicate that the target label does not describe the stimulus well. The SI reflects the extent to which the target label is perceived as a good description of the image, over and above distractor states or action labels. SI was computed by subtracting the mean rating given to selected distractor labels from the rating given to the target label, and taking the mean of these values across all stimulus ratings. SI values range between – 5 and 5. Negative values indicate that the target label received a lower score than the distractor labels taken together. Conversely, positive values signify that the target label received a higher rating compared to distractor labels taken together. The SI+ was obtained by subtracting the *highest* distractor rating from the rating given to the target label, and taking the mean of these values across all stimulus ratings. SI+ is a score ranging between – 5 and 5, whereby negative values indicate that distractor labels were given higher ratings than the target label, whilst positive values indicate that the target label received a higher rating than the distractor with the highest rating. The SI and SI+ are more conservative scores than the QI, as they take into account the discrepancy between ratings of intended and unintended labels. Values of SI and SI+ close to 0 indicate that the target label is not perceived to be a better description of the stimulus than the distractor labels. The CR consists of the proportion of participants who selected the target label to describe the stimulus, regardless of the quality rating given. CR scores range between 0% to 100%, whereby 0% indicates that the target label was never selected to describe the image, whilst 100% indicates that the target label was always selected to describe the image. The CR+ is the proportion of participants who gave the target label the highest quality rating of all labels. CR+ was calculated by assigning a score of 1 to those stimuli whose target label received the highest quality rating. Whenever a distractor obtained a quality rating equal to or higher than the target, a score of 0 was assigned. CR+ scores of 0% indicate that the target label was never rated higher than distractor labels when describing the image. CR+ scores of 100% indicate that the target label always received the highest rating, compared to distractor labels, when describing the image. All five scores are presented for each stimulus in Table 3 in the Appendix.

**Fig. 2:**
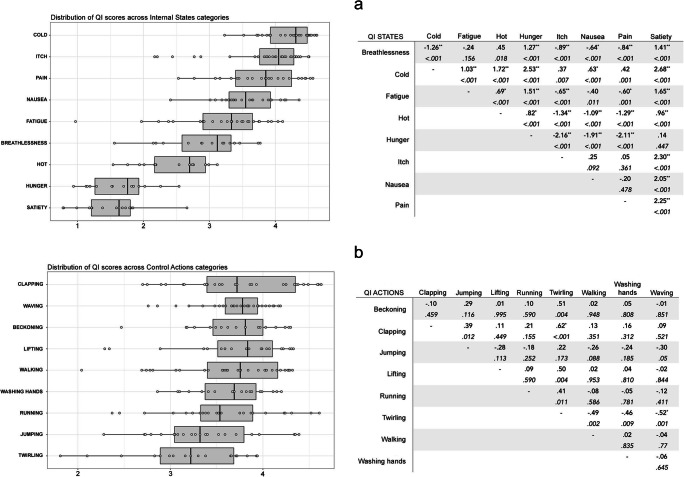
Distribution of Quality Index (QI) scores across different Stimulus Categories of Internal States (**a**) and Control Actions (**b**). The boxplots for each state and action are presented. Individual stimuli are plotted as single data points over the boxplot. Both graphs are presented alongside tables of post hoc *t* tests showing the mean difference (row - column) for each pair of Internal States (panel a) and Control Actions (panel b). *Asterisks* denote statistical significance at alpha level of .001 (**) and .05 (*) after Bonferroni corrections. The *p* value before Bonferroni correction is reported in italics below the mean difference value

Whole-sample analyses revealed that QI scores were higher for action stimuli (*M* = 3.62, *SD* = .55) than for internal state stimuli (*M* = 3.22, *SD* = 1.02) [*t*(421) = – 5.09, *p* < .001]. Separate ANOVAs were conducted for QI of internal states and QI of control actions, with Stimulus Category (all internal state/action stimulus categories) and Actor Sex (male, female) as IVs. For the internal states, a significant main effect of Stimulus Category [*F* (17, 184) = 62.97, *p* < .001, η^2^ = .73] was found. Cold received the highest QI (*M* = 4.2, *SD* = .35). Conversely, the lowest QI was attributed to hunger (*M* = 1.67, *SD* = .44) (Fig. 2a). Post hoc *t* tests were conducted across all pairs of states with Bonferroni corrections and are shown in Fig. 2a. Similarly, the ANOVA for the action stimuli resulted in a significant main effect of Stimulus Category [*F* (17, 203) = 2.63, *p* = .009, η^2^ = .09]. Clapping stimuli had the highest QI (*M* = 3.8, *SD* = .59), while the mean QI for twirling was the lowest of the action stimulus set (*M* = 3.18, *SD* = .60) (Fig. 2b). Post hoc *t* tests comparing all pairs of actions, with Bonferroni corrections, and are shown in Fig. 2b). Actor Sex did not contribute to variations in QI for either internal states [*F* (17, 184) = .06, *p* = .81] or control actions [*F* (17, 203) = .49, *p* = .48], and did not interact with Stimulus Category in either internal states [*F*(17, 184) = 1.75, *p* = .09] or control actions [*F*(17, 203) = 1.38, *p* = .21].

SI scores were higher for action stimuli (*M* = 3.01, *SD* = .86) than internal state stimuli (*M* = 1.90, *SD* = 1.84) [*t*(421) = – 8.08, *p* < .001)]. Again, separate ANOVAs were conducted for the action and the internal states stimulus sets, with SI as the DV and Stimulus Category and Actor Sex as IVs.

**Fig. 3:**
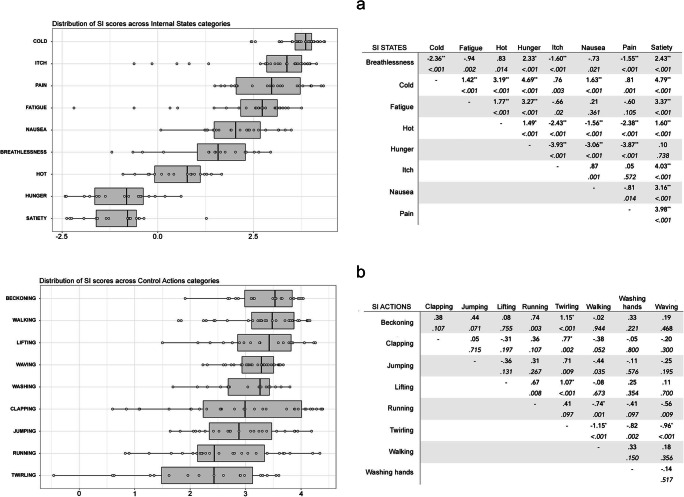
Distribution of Specificity Index (SI) scores across different Stimulus Categories of Internal States (**a**) and Control Actions (**b**). The boxplots for each state and action are presented. Individual stimuli are plotted as single data points over the boxplot. Both graphs are presented alongside tables of post hoc *t* tests showing the mean difference (row - column) for each pair of Internal States (panel a) and Control Actions (panel b). *Asterisks* denote statistical significance at alpha level of .001 (**) and .05 (*) after Bonferroni corrections. The *p* value before Bonferroni corrections is reported in italics below the mean difference value

For the internal states, the main effect of Stimulus Category was significant [*F* (17, 184) = 63.197, *p* < .001, η^2^ = .73]. Cold had the highest SI (*M* = 3.71, *SD* = .50), while SI was lowest for satiety (*M* = – 1.07, *SD* = .91) (Fig. 3a). Post hoc *t* tests for Stimulus Category using Bonferroni corrections are shown in Fig. 3a. There was a significant main effect of Actor Sex [*F* (17, 184) = 5.04, *p* = .02, η^2^ = .03], whereby SI scores were higher for stimuli depicted by female actors (*M* = 1.99, *SD* = 1.77) than those portraying male actors (*M* = 1.81, *SD* = 1.90). Actor Sex did not interact significantly with Stimulus Category [*F* (17, 184) = 1.96, *p* = .054]. For the action stimulus set, a significant main effect of Stimulus Category [*F* (17, 203) = 4.76, *p* < .001, η^2^ = .16] was observed. Beckoning and twirling had the highest (*M* = 3.36, *SD* = .58) and lowest (*M* = 2.21, *SD* = 1.14) SIs, respectively (Fig. 3b). Post hoc *t* tests using Bonferroni corrections were conducted on Stimulus Category and are reported in Fig. 3b. The main effect of Actor Sex [*F* (17, 203) = .41, *p* = .52] and the interaction between Actor Sex and Stimulus Category [*F* (17, 203) = 1.67, *p* = .11] were non-significant.

**Fig. 4:**
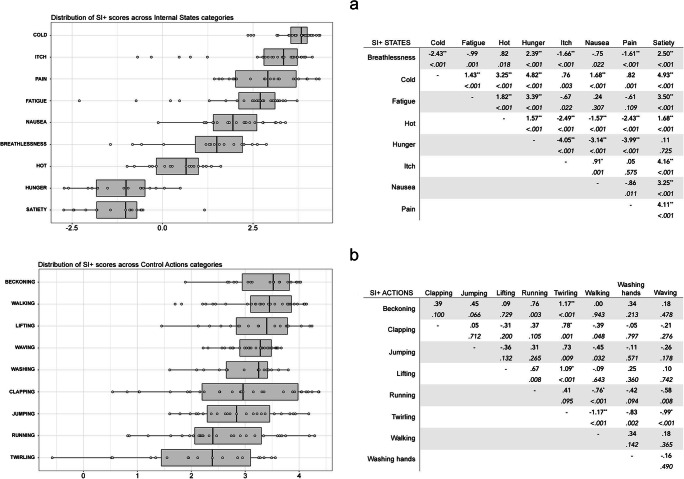
Distribution of Max-distractor Specificity Index (SI+) scores across different Stimulus Categories of Internal States (**a**) and Control Actions (**b**). The boxplots for each state and action are presented. Individual stimuli are plotted as single data points over the boxplot. Both graphs are presented alongside tables of post hoc *t* tests showing the mean difference (row - column) for each pair of Internal States (panel a) and Control Actions (panel b). *Asterisks* denote statistical significance at alpha level of .001 (**) and .05 (*) after Bonferroni corrections. The *p* value before post hoc corrections is reported in italics below the mean difference value

SI+ was significantly higher for action stimuli (*M* = 2.99, *SD* = .87) than for internal state stimuli (*M* = 1.82, *SD* = 1.89) [*t*(421) = – 8.21, *p* < .001]. Separate ANOVAs were conducted for the action and internal state stimulus sets, with Stimulus Category and Actor Sex as IVs and SI+ scores as the DV. A significant main effect of Stimulus Category was found for internal state stimuli [*F* (17, 184) = 63.797, *p* < .001, η^2^ = .735]. Cold and Satiety had the highest (*M* = 3.68, *SD* = .51) and lowest (*M* = – 1.25, *SD* = .95) SI+, respectively (Fig. 4a). Bonferroni-corrected post hoc *t* tests were conducted on all the levels of Stimulus Category and are reported in Fig. 4a. A significant main effect of Actor Sex was found [*F* (17, 184) = 5.35, *p* < .05, η^2^ = .03] whereby stimuli depicting female actors (*M* = 1.93, *SD* = 1.82) received slightly higher SI+ score than those depicting male actors (*M* = 1.73, *SD* = 1.96). Actor Sex did not interact with Stimulus Category [*F* (17, 184) = 1.93, *p* = .06]. The ANOVA for SI+ scores of action stimuli resulted in a significant main effect of Stimulus Category [*F* (17, 203) = 4.87, *p* < .001, η^2^ = .16]. Beckoning was the category to receive the highest SI+ scores (*M* = 3.34, *SD* = .58), whilst Twirling received the lowest SI+ scores (*M* = 2.17, *SD* = 1.16) (Fig. 4b). Post hoc *t* tests for each pair of action categories were conducted, using Bonferroni corrections (Fig. 4b). Actor sex did not contribute to variation in total SI+ scores [*F* (17, 203) = .38, *p* = .54] and did not interact with Stimulus Category [*F* (17, 203) = 1.65, *p* = .11].

**Fig. 5:**
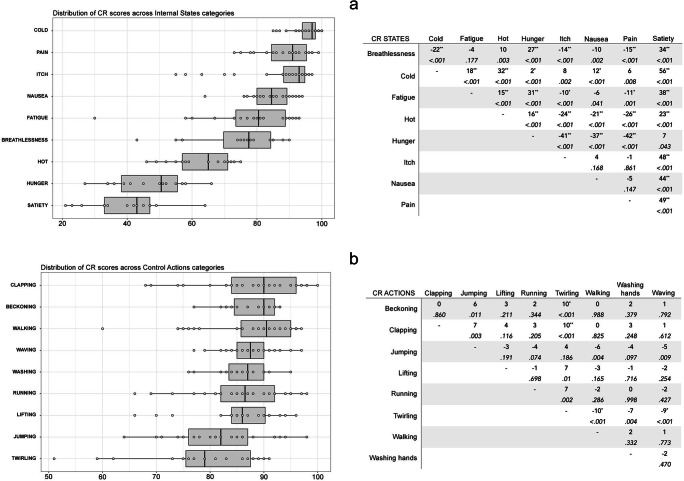
Distribution of Choice Rate (CR) scores across different Stimulus Categories of Internal States (Panel a) and Control Actions (Panel b). The boxplots for each state and action are presented. Individual stimuli are plotted as single data points over the boxplot. Both graphs are presented alongside tables of post-hoc t-tests showing the mean difference (row - column) for each pair of Internal States (Panel a) and Control Actions (Panel b). Asterisks denote statistical significance at alpha level of .001 (**) and .05 (*) after Bonferroni corrections. The p value before post hoc corrections is reported in italics below the mean difference value

CR scores showed that participants selected the target label to describe action stimuli (*M* = 86%, *SD* = 8%) significantly more often than they did to describe internal states (*M* = 77%, *SD* = 20%) [*t*(421) = -6.10, *p* < .001]. ANOVAs were computed for CR scores of internal states and control actions separately, with Stimulus Category and Actor Sex as IVs. For the internal states, a significant main effect of Stimulus Category [*F* (17, 184) = 73.89, *p* < .001, η^2^ = .76] was observed. Cold was the state with the highest CR (96%), whilst satiety had the lowest CR (40%) (Fig. 5a). Post hoc *t* tests using Bonferroni corrections are displayed in Fig. 5a. There was no main effect of Actor Sex [*F* (17, 184) = .09, *p* = .93] or interaction between Actor Sex and Stimulus Category [*F* (17, 184) = 1.08, *p* = .38]. The ANOVA for the action stimuli resulted in a significant main effect of Stimulus Category [*F* (17, 203) = 4.03, *p* < .001, η^2^ = .14]. Clapping had the highest CR (89%), whereas twirling was the action with the lowest CR (78%) (Fig. 5b). Post hoc *t* tests with Bonferroni correction across all categories are shown in Fig. 5b. Actor Sex did not contribute to variations of CR [*F* (17, 203) = 1.25, *p* = .26] or interact with Stimulus Category [*F* (17, 203) = .83, *p* = .58].

**Fig. 6:**
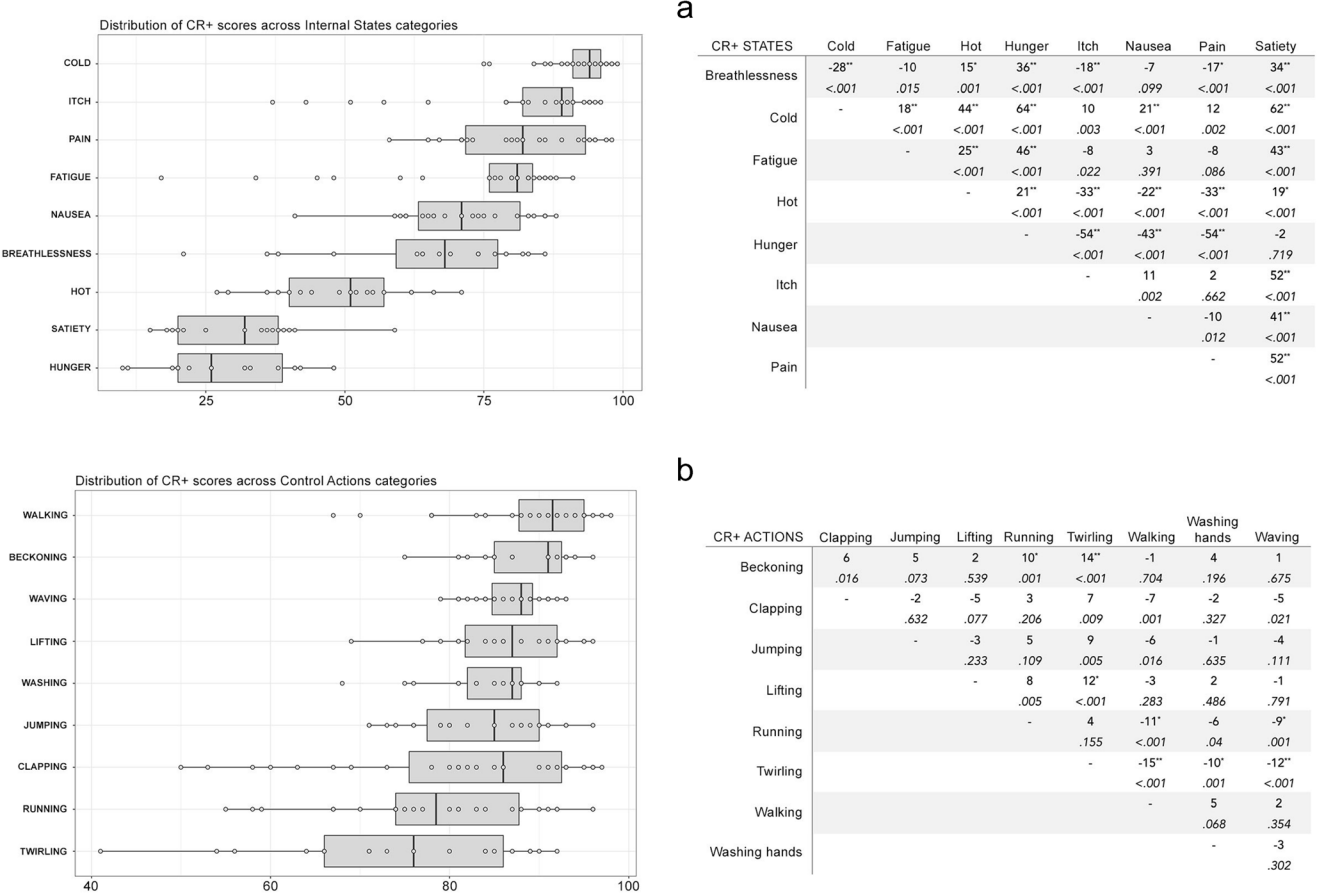
Distribution of High-quality Choice Rate (CR+) scores across different Stimulus Categories of Internal States (**a**) and Control Actions (**b**). The boxplots for each state and action are presented. Individual stimuli are plotted as single data points over the boxplot. Both graphs are presented alongside tables of post hoc *t* tests showing the mean difference (row - column) for each pair of Internal States (panel a) and Control Actions (panel b). *Asterisks* denote statistical significance at alpha level of .001 (**) and .05 (*) after Bonferroni corrections. The alpha level before post hoc corrections is reported in italics below the mean difference value

Finally, CR+ scores for action stimuli (*M* = 84%, *SD* = 10%) were significantly higher than CR+ scores for internal state stimuli (*M* = 69%, *SD* = 25%) [*t*(421) = – 8.56, *p* < .001]. Once again, separate ANOVAs were conducted for the CR+ scores of action and internal states stimuli, with Stimulus Category and Actor Sex as IVs. For the internal states, a main effect of Stimulus Category was found [*F* (17, 184) = 61.80, *p* < .001, η^2^ = .73]. Cold stimuli received the highest (*M* = 92%, *SD* = 6%) CR+ scores, whilst Satiety stimuli had the lowest CR+ scores (*M* = 30%, *SD* = 12%) (Fig. 6a). Bonferroni corrected post-hoc t-tests across all pairs of states are shown in Fig. 6a. A significant main effect of Actor Sex was found [*F* (17, 184) = 5.77, *p* < .05, η^2^ = .03] whereby internal state stimuli depicting female actors (*M* = 70%, *SD* = 24%) received slightly higher CR+ scores than those depicting male actors (*M* = 67%, *SD* = 25%). Finally, Actor Sex did not interact significantly with Stimulus Category [*F* (17, 184) = 1.62, *p* = .12]. The ANOVA for the action stimuli returned a significant main effect of Stimulus Category [*F* (17, 203) = 6.27, *p* < .001, η^2^ = .198], whereby Walking and Twirling received the highest (*M* = 89%, *SD* = 8%) and lowest (*M* = 75%, *SD* = 14%) CR+ scores, respectively (Fig. 6b). Post-hoc t-tests with Bonferroni corrections across all pairs of actions are shown in Fig. 6b. The effect of Actor Sex on variations of CR+ scores did not reach statistical significance [*F* (17, 203) = .41, *p* = .52]. Likewise, Actor Sex did not interact with Stimulus Category [*F* (17, 203) = 1.71, *p* = .10].

To investigate the effect of observer gender on the evaluation of internal state stimuli, separate ANOVAs were conducted for the five recognition indices with Stimulus Category (all the internal states) and Observer Gender (female, male) as factors. The ANOVA with QI as DV did not reveal a main effect of Observer Gender [*F*(17, 386) = .57, *p* = .45, η^2^ = .001]. Moreover, Observer Gender did not interact significantly with Stimulus Category [*F*(17, 386) = .77, *p* = .63, η^2^ = .02]. The ANOVA for SI scores returned a main effect of Stimulus Category [*F*(17, 386) = 111.41, *p* < .001, η^2^ = .698], and a main effect of Observer Gender [*F*(17, 386) = 10.74, *p* = .001, η^2^ = .03], whereby female observers (*M* = 2.09, *SD* = 1.83) had higher SI indices than male observers (*M* = 1.76, *SD* = 1.90). Observer Gender did not interact significantly with Stimulus Category [*F*(17, 386) = .57, *p* = .80, η^2^ = .01]. The ANOVA for SI+ scores revealed a main effect of Stimulus Category [*F*(17, 386) = 112.55, *p* < .001, η^2^ = .70], and a main effect of Observer Gender [*F*(17, 386) = 11.25, *p* = .001, η^2^ = .03], with female observers (*M* = 2.03, *SD* = 1.88) having higher SI+ indices than male observers (*M* = 1.67, *SD* = 1.95), but no interaction between Observer Gender and Stimulus Category [*F*(17, 386) = .54, *p* = .82, η^2^ = .01]. The ANOVA for CR scores resulted in a main effect of Stimulus Category [*F*(17, 386) = 123.91, *p* < .001, η^2^ = .72], and a main effect of Observer Gender [*F*(17, 386) = 7.13, *p* = .008, η^2^ = .02], where female observers (*M* = 95.53, *SD* = 4.65) had slightly higher CR scores than males (*M* = 94.87, *SD* = 4.87). The interaction between the two factors was non-significant [*F*(17, 386) = .68, *p* = .708, η^2^ = .01]. Finally, the ANOVA for CR+ scores returned a main effect of Stimulus Category [*F*(17, 386) = 108.03, *p* < .001, η^2^ = .691], and a main effect of Observer Gender [*F*(17, 386) = 5.66, *p* = .02, η^2^ = .01], with higher CR+ scores in female observers (*M* = 72.59, *SD* = 23.70) than male observers (*M* = 69.34, *SD* = 24.50), but no interaction between Observer Gender and Stimulus Category [*F*(17, 386) = .56, *p* = .812, η^2^ = .01].

#### Confusion across stimulus categories

**Fig. 7:**
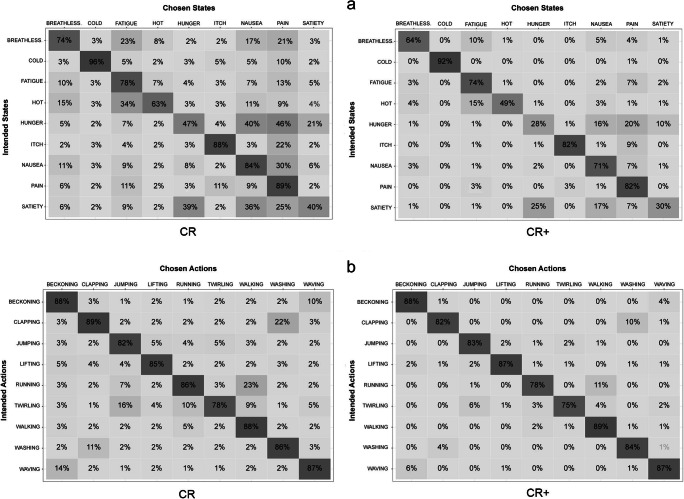
Confusion matrixes showing the proportion of the time that each label was used to describe stimuli of each intended state (Choice Rate (CR) matrix) and the proportion of the time that each label was given the highest quality rating to describe stimuli of each intended state (High-quality Choice Rate (CR+) matrix). Confusion matrices are presented separately for Internal States (panel a) and Control Actions (panel b)

In order to determine which states/actions were confused with each other, confusion scores were created based on CR and CR+ scores. Confusion matrices were created whereby each row corresponds to the intended state or action portrayed by the actor, and each column represents the proportion of times each state or action label was selected regardless of quality rating in the CR matrix, and the proportion of times each state or action label was given the highest quality rating in the CR+ matrix. Among the internal states, some categories were particularly confused with others; Hunger stimuli were often rated as depicting Pain (CR = 46%; CR+ = 20%) and Nausea (CR = 40%; CR+ = 16%), Satiety stimuli were also rated as depicting Hunger (CR = 39%; CR+ = 25%) and Nausea (CR = 36%; CR+ = 17%), and Nausea stimuli were often rated as depicting Pain (CR = 30%; CR+ = 7%) (Fig. 7a). On the other hand, the confusion matrix for action stimuli revealed lower levels of confusion (i.e. target actions were less often labelled as non-target actions). Clapping stimuli were sometimes labelled as depicting Washing Hands (CR = 22%; CR+ = 10%), Running stimuli were also rated as depicting Walking (CR = 23%; CR+ = 11%), Twirling stimuli were sometimes rated as depicting Jumping (CR = 16%; CR+ = 6%), Waving stimuli were also rated as depicting Beckoning (CR = 14%; CR+ = 6%), and Beckoning stimuli were occasionally labelled as depicting Waving (CR = 10%; CR+ = 4%) (Fig. 7b).

## Discussion

The current report presents the creation and validation of the ISSI database, a novel stimulus set of 423 static images representing non-affective internal bodily states and control actions. Each stimulus is presented alongside a range of indices from the second stage of validation, representing the quality and specificity of depiction, and the extent to which each stimulus was recognised as the intended state or action. Confusion matrices of internal states and control actions are also included to provide an indication of which states and which actions tend to be confused with each other. The stimuli are freely available to researchers for their use in scientific research and can be downloaded from the Insulab website (https://www.insulab.uk).

Overall, 77% (*Ra*: 40–96%) of participants selected the intended label to describe the internal state stimuli, and 86% (*Ra:* 78–89%) of participants selected the intended label to describe action stimuli. When observer gender was considered, female observers gave higher ratings and were more likely to select the intended label for the stimuli compared to male observers. Within the internal state stimulus set, there was high variability between stimulus categories in terms of quality and specificity of depiction, and proportion of participants selecting the target state, with the pattern of results across different indices being relatively consistent. Satiety was the most difficult state to recognise and discriminate from other states, followed by hunger. Hunger and satiety stimuli were given fairly low quality (QI) scores, with the majority being given a mean score below 2 (‘*Poor’* on the rating scale), and negative specificity (SI and SI+) scores, indicating that distractor labels were often judged to be better descriptors of the stimulus than the target label. Similarly, CR and CR+ scores were often under 50%, indicating that the target label was selected to describe the stimulus (CR), or as the best descriptor of the stimulus (CR+), less than half of the time. Other internal state categories, however, were given high quality and specificity ratings, and the intended label was selected frequently. The vast majority of cold, itch, pain, fatigue and nausea stimuli, for example, were given QI scores above 3, positive SI/SI+ scores, and CR/CR+ scores above 70%. While there is therefore variability across internal state categories and individual stimuli, all stimuli rated in the second validation stage have been retained in the final stimulus set, in order for researchers to select stimuli according to their own research requirements. While we would recommend using stimuli with high quality, specificity and choice rate scores where studies require stimuli that have been validated and are recognisable by typical participants as their intended state, the range of recognition scores also allows for the study of ambiguous stimuli, or internal states that are easily confused. Notably, action stimuli were consistently recognised better than internal state stimuli, and there was less variability among different action stimulus categories in terms of quality and specificity ratings, and the extent to which the intended label was selected to describe the stimuli. Variability was also observed across actors, both in terms of quality of depiction and recognisability of the stimuli produced. Individual differences in the ability to produce recognisable non-affective internal states are expected, and elucidating the predictors of such differences should be investigated in future research. Previous works on facial expressions of emotion indicates, for example, that autistic individuals produce less typical emotional expressions compared to neurotypical individuals (e.g., Brewer et al., 2016; Langdell, 1981). Further research is needed to elucidate whether a similar pattern is observed for the expression of interoceptive states.

It is likely that internal states were recognised less well than action stimuli due to the associations and similarities between internal states giving rise to greater confusability. In particular, there is an over-representation of gastric internal signals in the current stimulus set (i.e. nausea, hunger, satiety), which could be responsible for lower specificity scores and choice rates for these stimulus categories. Actors frequently expressed these internal states by placing their hands on or around the abdomen, likely making these stimuli difficult to differentiate. Crucially, despite variability in the low-level visual features of the stimuli within state categories, there was consistency across actors’ depictions of states, and visual cues were often in line with those that would be expected based on the location at which states are perceived within the body (e.g., the abdomen). Notably, recognition scores are likely to be dramatically increased if fewer gastric response options are available to participants (e.g., researchers could include nausea, hunger, and satiety under the same umbrella term ‘gastric discomfort’); in the current validation task, the availability of all target labels may have led to more conservative recognition estimates, while in a two-alternative forced choice task where stimuli must be labelled as either cold or satiety, for example, it is likely that participants would perform near ceiling, as the visual cues associated with these states are highly distinct. Indeed, in tasks assessing affective emotion recognition, recognition accuracy is improved by having fewer available response options, or less confusable response options in alternative forced choice tasks. For example, angry facial expressions were less likely to be labelled as depicting anger in a task where response options included “anger”, “frustration”, and “contempt” than when fewer closely related response options were available (Russell, 1993). Similarly, recognition of happiness expressions (which often shows ceiling effects even in those with difficulties recognising other facial expressions) has been found to be impaired in those with emotion processing impairments (alexithymia) when stimuli depicting pain are included in the recognition task, likely due to painful expressions sharing perceptual characteristics with happy expressions (Brewer et al., 2015). In contrast, action stimulus categories were more distinct from each other in their associated behavioural cues, and therefore less confusable with each other.

Naturalness of expression may have also played a role in the disparity of recognition scores among stimulus categories. Although visual behavioural expressions of states such as hunger and satiety, such as rubbing the abdomen (hunger and satiety) or exhaling heavily (satiety), do occur, they may be less spontaneous than behavioural expressions of other states such as feeling cold (e.g. rubbing one’s arms) or feeling itchy (e.g. scratching one’s skin). This may be due to the behavioural responses to cold and itch serving a purpose to reduce the internal state, and thus being performed more often, rather than serving a more communicative purpose and therefore only being used in social situations, and potentially less frequently. Similarly, actions that are performed with a more communicative purpose may be more frequently accompanied by a verbal description (e.g. stating ‘I’m so hungry’ while rubbing one’s abdomen), reducing the requirement for an observer to recognise the visual signals. It is worth noting that facial expressions of affective states can be either spontaneous or posed for communicative purposes, and these tend to differ in their visual features, such as onset time, duration, and amplitude of physical facial movement (Schmidt et al., 2006; Valstar et al., 2006). It is likely that spontaneous and posed/communicative expressions of non-affective internal states also differ, and the extent to which they differ may vary across internal states. It is possible that actors’ depictions of internal states were therefore more recognisable for states where spontaneous and posed expressions of the state are more similar, making the actors’ depiction more ecologically valid. For states which either are infrequently expressed, or for which spontaneous expressions differ greatly from posed expressions, actors’ depictions may have been less recognisable. Notably, the communicative value of individual internal states may also vary across different cultures. Future research is needed to examine cross-cultural influences on the expression and recognition of internal states.

Moreover, the expression of certain internal states is likely to be multidimensional, with expressions including a combination of visual (e.g. kinematic), auditory (e.g. vocal) and contextual cues. Recognition of internal states in others may, therefore, be greatly improved by the addition of vocal cues, body movement, or contextual information. When observing an individual rubbing their abdomen, for example, contextual information might be necessary in order to interpret the action accurately as a sign of hunger (e.g. it is lunch time and we are in a queue to buy food), rather than a sign of satiety (e.g. we just ate a large meal). Future research is needed to elucidate whether some states rely more than others on visual cues for their expression, and what type of cues are necessary for their recognition. The availability of stimuli depicting states that are easily confused with each other in this stimulus set will make it possible to address these research questions. Notably, research into the perception and recognition of non-affective internal states in others will pose new methodological challenges, in part complementary to those faced when studying the perception of internal states in the self. On one hand, some internal states (e.g. itch, fatigue) are associated with visual cues but are difficult to measure objectively, potentially making study of these states easier in relation to others than to the self. Conversely, some internal states, such as cardiac signal changes, are easy to objectively assess in the individual, but are not accompanied by visual cues, making them difficult to observe in others.

Another crucial aspect to consider is the relative role of facial and bodily information in participants’ recognition of the current stimulus set. Facial cues were not obscured from the stimuli in either validation stage, as both facial expressions and postural cues are likely to be important for conveying internal states, and full body postures were deemed to be the most ecologically valid. It is possible, however, that facial and body information are recognisable in isolation, or that the relative contribution of facial and body cues to state recognition varies across internal states. As emotional cues are particularly expressed by the face (Adolphs, 1999; Frith, 2009), and it may be possible to experience affective and non-affective states simultaneously, interference effects from emotional cues may be especially evident when facial cues are present. While we note that stimuli have only been validated with integrated facial and body cues, it is of course possible for future work to investigate recognition from distinct regions of the stimuli, for example by separating or manipulating facial and body cues.

Crucially, the theoretical distinction between affective (emotional) and interoceptive states is not clear-cut. Here we refer to interoceptive states as internal bodily sensations beyond the affective domain. With this, we do not imply that emotions and interoceptive states are necessarily separate entities. On the contrary, according to the leading model of emotion perception, interoception is a fundamental component of emotional experience, which derives from sensory and affective experiences in combination with contextual cues (Schachter & Singer, 1962). However, it is common in the literature to find emotion processing and interoception treated as separate components. Similarly, some states, such as pain, seem to be considered as both emotional and interoceptive, with Craig describing pain as a ‘homeostatic emotion’ due to its sensory component alongside a motivational drive to re-establish the body’s homeostasis (Craig, 2003b). This definition could arguably be applied to a number of interoceptive states. Future work is needed to assess whether individuals process affective and interoceptive states in others differently. To this end, the call for stimuli depicting internal sensations beyond the affective domain is even more critical. Going forward, it is important that categories of internal states are clearly defined, both theoretically and operationally.

In conclusion, the ISSI stimulus set will allow, for the first time, the investigation of humans’ ability to recognise non-affective internal states in others. There are opportunities for investigating this basic process, for example the role of contextual cues and the contribution of facial and body postural cues to recognition, as well as for investigating correlates of individual differences in this ability, the genetic and neural basis of recognition, developmental trajectories, and the relationship between psychopathology and recognition abilities. Less recognisable stimuli have not been eliminated from the database, as researchers are encouraged to select stimuli based on their specific needs and research questions. If the aim of the study is that of assessing the accuracy of internal state recognition, then we advise researchers to select stimuli with higher quality, specificity, and choice rates, as these offer greater validity. The availability of more ambiguous stimuli, however, will allow investigation of individual differences in interpretation, and the biasing role of additional cues, for example. Researchers using the ISSI stimuli are encouraged to report their stimulus selection process transparently, and may utilise the validation statistics in the ISSI database to do this.

## Appendix

**Table 3:** Recognisability, quality, specificity, and accuracy scores for each stimulus of the ISSI database. *RI* Recognisability Index, *QI* Quality Index, *SI* Specificity Index, *SI+*Maximum-Distractor Specificity Index, *CR* Choice Rate, *CR+*High-Quality Choice Rate. The stimulus name indicates, in order, the state/action displayed, the actor’s gender (M or F), the actor’s identifier (1–4), and the exemplar (1–4)

STIMULUS	RI (%)	QI	SI	SI+	CR (%)	CR+ (%)
BREATHLESSNESS_M1_1	20	3.46	2.28	2.19	85	77
BREATHLESSNESS_M1_2	10	2.68	1.33	1.29	74	63
BREATHLESSNESS_M2_1	45	2.30	– 0.40	– 0.58	57	38
BREATHLESSNESS_M2_2	25	2.88	1.51	1.43	78	74
BREATHLESSNESS_M3_1	65	2.19	– 0.65	– 0.83	55	36
BREATHLESSNESS_M3_2	15	3.04	1.76	1.70	75	69
BREATHLESSNESS_M4_1	5	1.56	– 1.20	– 1.44	43	21
BREATHLESSNESS_M4_2	15	3.77	2.64	2.62	88	82
BREATHLESSNESS_F1_1	25	3.74	2.95	2.87	90	86
BREATHLESSNESS_F1_2	25	3.20	2.31	2.25	79	79
BREATHLESSNESS_F2_1	45	2.15	0.15	0.03	57	48
BREATHLESSNESS_F2_2	30	3.31	2.52	2.48	84	83
BREATHLESSNESS_F3_1	50	3.22	1.64	1.57	78	67
BREATHLESSNESS_F3_2	45	3.22	1.34	1.20	76	64
BREATHLESSNESS_F4_1	25	3.38	2.03	1.97	85	74
BREATHLESSNESS_F4_2	25	2.89	1.49	1.33	77	64
COLD_M1_1	100	4.51	4.36	4.35	98	99
COLD_M1_2	90	4.36	3.93	3.87	99	95
COLD_M1_3	100	4.08	3.71	3.69	95	96
COLD_M1_4	60	3.72	3.17	3.16	92	90
COLD_M2_1	90	4.23	3.86	3.83	94	95
COLD_M2_2	55	3.74	3.18	3.16	87	86
COLD_M2_3	100	4.49	4.25	4.25	98	98
COLD_M2_4	100	4.36	3.97	3.94	96	92
COLD_M3_1	95	4.50	4.05	4.02	99	93
COLD_M3_2	70	3.91	3.24	3.19	93	87
COLD_M3_3	95	4.48	3.93	3.86	99	93
COLD_M3_4	100	4.15	3.67	3.66	94	89
COLD_M4_1	100	4.62	4.01	3.97	100	96
COLD_M4_2	60	3.72	2.44	2.36	89	76
COLD_M4_3	90	4.32	3.70	3.66	98	92
COLD_M4_4	100	4.53	4.07	4.03	98	96
COLD_F1_1	85	4.30	3.51	3.49	97	94
COLD_F1_2	80	3.87	3.52	3.50	95	91
COLD_F1_3	85	3.93	3.75	3.70	98	91
COLD_F1_4	95	3.80	3.61	3.59	92	94
COLD_F2_1	95	4.59	4.18	4.16	98	97
COLD_F2_2	95	4.29	4.09	4.08	100	99
COLD_F2_3	95	4.32	3.87	3.84	98	97
COLD_F2_4	95	4.55	4.34	4.32	100	98
COLD_F3_1	100	4.32	3.63	3.56	97	93
COLD_F3_2	100	4.47	3.98	3.94	97	95
COLD_F3_3	100	4.63	4.17	4.12	100	97
COLD_F3_4	95	4.23	3.71	3.67	97	92
COLD_F4_1	60	4.27	3.96	3.93	96	95
COLD_F4_2	100	4.39	4.01	3.99	97	96
COLD_F4_3	55	3.23	2.55	2.52	86	84
COLD_F4_4	75	3.52	2.46	2.44	85	75
FATIGUE_M1_1	45	0.97	– 2.19	– 2.30	30	17
FATIGUE_M1_2	65	2.86	2.71	2.71	77	83
FATIGUE_M1_3	70	1.97	– 0.62	– 0.73	58	34
FATIGUE_M1_4	65	2.45	0.32	0.22	67	48
FATIGUE_M2_1	70	2.90	2.31	2.25	73	76
FATIGUE_M2_2	100	3.18	2.64	2.64	79	80
FATIGUE_M2_3	60	3.05	1.47	1.29	77	60
FATIGUE_M3_1	80	3.94	3.76	3.72	91	86
FATIGUE_M3_2	45	2.38	0.52	0.47	67	45
FATIGUE_M3_3	90	3.89	3.27	3.24	92	91
FATIGUE_M4_1	95	4.11	3.06	2.99	93	86
FATIGUE_M4_2	90	4.04	2.75	2.69	93	81
FATIGUE_M4_3	70	3.76	2.12	2.06	90	76
FATIGUE_M4_4	90	3.49	2.63	2.60	89	80
FATIGUE_F1_1	70	3.34	3.08	3.03	80	81
FATIGUE_F1_2	95	3.75	3.41	3.40	90	88
FATIGUE_F1_3	65	2.91	2.82	2.80	75	83
FATIGUE_F2_1	80	3.27	2.55	2.48	81	78
FATIGUE_F2_2	95	3.33	3.20	3.19	79	85
FATIGUE_F2_3	100	3.67	3.34	3.33	82	87
FATIGUE_F2_4	95	2.92	2.57	2.53	73	77
FATIGUE_F3_1	100	3.42	3.13	3.12	81	84
FATIGUE_F3_2	50	2.23	1.79	1.75	60	64
FATIGUE_F3_3	100	3.51	3.10	3.07	82	83
FATIGUE_F4_1	95	3.58	3.19	3.17	82	83
FATIGUE_F4_2	95	3.60	2.82	2.77	88	83
HOT_M1_1	10	2.70	0.42	0.27	72	42
HOT_M1_2	35	2.76	1.40	1.35	68	62
HOT_M2_1	45	2.70	0.86	0.74	73	54
HOT_M2_2	30	2.18	0.10	0.06	55	52
HOT_M3_1	5	2.01	– 0.60	– 0.78	58	27
HOT_M3_2	20	1.94	– 0.91	– 0.98	52	29
HOT_M4_1	25	2.94	0.29	0.21	70	44
HOT_M4_2	35	3.12	1.11	0.99	75	57
HOT_F1_1	60	2.54	0.96	0.87	65	55
HOT_F1_2	55	1.76	– 0.22	– 0.28	49	38
HOT_F2_1	20	2.19	– 0.08	– 0.17	59	40
HOT_F2_2	10	1.54	– 0.25	– 0.36	46	36
HOT_F3_1	45	2.17	0.88	0.80	57	51
HOT_F3_2	35	2.74	0.77	0.65	65	49
HOT_F4_1	65	2.99	1.28	1.21	71	66
HOT_F4_2	60	2.99	1.67	1.61	71	71
HOT_F4_3	45	2.94	1.22	1.12	71	62
HUNGER_M1_1	30	2.26	– 0.07	– 0.18	57	42
HUNGER_M1_2	40	2.54	0.61	0.49	66	48
HUNGER_M2_1	20	1.53	– 1.60	– 1.79	41	20
HUNGER_M2_2	25	1.52	– 1.53	– 1.79	45	20
HUNGER_M3_1	20	1.82	– 0.46	– 0.61	52	32
HUNGER_M3_2	25	1.93	– 0.87	– 1.07	58	26
HUNGER_M4_1	10	1.19	– 1.78	– 1.95	34	20
HUNGER_M4_2	10	1.13	– 2.42	– 2.72	36	10
HUNGER_F1_1	15	1.29	– 1.37	– 1.52	39	22
HUNGER_F1_2	25	0.94	– 2.39	– 2.60	27	11
HUNGER_F2_1	25	2.02	0.19	0.05	57	48
HUNGER_F2_2	15	1.85	– 0.76	– 0.95	52	26
HUNGER_F3_1	20	1.93	– 0.23	– 0.35	55	41
HUNGER_F3_2	15	1.15	– 1.92	– 2.06	34	19
HUNGER_F4_1	25	1.70	– 0.51	– 0.60	50	33
HUNGER_F4_2	25	1.85	– 0.43	– 0.53	51	38
ITCH_M1_1	70	3.31	2.68	2.62	83	82
ITCH_M1_2	100	4.23	3.37	3.31	96	89
ITCH_M1_3	90	3.74	2.81	2.77	91	79
ITCH_M2_1	95	4.38	3.84	3.83	96	94
ITCH_M2_2	95	4.38	3.98	3.95	93	93
ITCH_M2_3	100	4.50	3.58	3.55	97	90
ITCH_M2_4	100	4.32	3.92	3.91	94	96
ITCH_M3_1	100	3.88	2.95	2.92	90	86
ITCH_M3_2	85	3.86	3.20	3.18	88	88
ITCH_M3_3	65	2.17	– 0.62	– 0.69	55	37
ITCH_M3_4	80	2.87	0.82	0.77	73	57
ITCH_M4_1	75	4.10	3.61	3.61	95	90
ITCH_M4_2	75	4.00	2.84	2.79	93	82
ITCH_M4_3	95	3.91	2.87	2.84	89	79
ITCH_M4_4	85	4.01	3.06	3.02	92	82
ITCH_F1_1	95	4.31	3.91	3.84	94	90
ITCH_F1_2	95	4.29	3.97	3.95	94	94
ITCH_F1_3	95	4.25	3.59	3.59	95	90
ITCH_F2_1	100	4.35	3.77	3.71	96	91
ITCH_F2_2	85	4.14	3.66	3.63	93	89
ITCH_F2_3	95	4.09	3.73	3.73	94	94
ITCH_F2_4	85	3.69	3.36	3.33	90	91
ITCH_F3_1	100	3.96	3.59	3.56	90	89
ITCH_F3_2	65	2.44	0.50	0.46	63	51
ITCH_F3_3	95	4.16	3.84	3.84	95	95
ITCH_F3_4	95	3.83	3.38	3.36	94	91
ITCH_F4_1	100	4.51	4.15	4.14	95	96
ITCH_F4_2	75	2.77	1.33	1.24	70	65
ITCH_F4_3	55	2.21	– 0.15	– 0.31	58	43
ITCH_F4_4	95	4.11	3.14	3.07	88	83
NAUSEA_M1_1	60	3.09	1.45	1.41	77	66
NAUSEA_M1_2	75	3.37	1.60	1.51	80	64
NAUSEA_M1_3	80	3.92	2.40	2.31	88	74
NAUSEA_M2_1	60	3.40	1.92	1.84	82	71
NAUSEA_M2_2	85	3.01	1.30	1.21	76	61
NAUSEA_M2_3	50	3.33	1.53	1.39	82	61
NAUSEA_M3_1	80	3.89	2.51	2.41	93	77
NAUSEA_M3_2	50	3.29	1.30	1.20	81	61
NAUSEA_M4_1	90	4.08	2.36	2.27	88	73
NAUSEA_M4_2	85	3.25	1.23	1.15	79	65
NAUSEA_M4_3	65	3.49	1.26	1.15	84	60
NAUSEA_M4_4	80	3.81	2.15	2.04	90	71
NAUSEA_F1_1	40	2.41	0.08	– 0.12	64	41
NAUSEA_F1_2	65	3.36	1.92	1.85	80	66
NAUSEA_F2_1	50	3.19	1.91	1.80	80	68
NAUSEA_F2_2	75	3.61	2.82	2.78	86	83
NAUSEA_F2_3	60	3.65	2.63	2.55	85	81
NAUSEA_F2_4	70	4.08	3.33	3.26	94	88
NAUSEA_F3_1	75	3.30	1.48	1.41	76	59
NAUSEA_F3_2	95	4.36	3.49	3.40	93	86
NAUSEA_F3_3	50	3.66	2.32	2.25	86	75
NAUSEA_F4_1	85	3.94	2.82	2.77	89	84
NAUSEA_F4_2	75	4.14	3.16	3.12	92	86
NAUSEA_F4_3	70	4.16	3.15	3.14	91	86
PAIN_M1_1	85	3.94	3.02	2.98	91	86
PAIN_M1_2	100	4.35	3.79	3.76	97	95
PAIN_M1_3	100	4.57	4.33	4.33	97	97
PAIN_M1_4	80	3.86	3.13	3.08	93	82
PAIN_M2_1	90	3.45	2.04	1.98	85	71
PAIN_M2_2	100	3.85	2.86	2.81	90	82
PAIN_M2_3	95	3.65	1.92	1.84	88	72
PAIN_M2_4	65	3.42	1.75	1.65	83	67
PAIN_M3_1	85	4.21	3.64	3.61	96	92
PAIN_M3_2	100	4.53	4.00	4.00	97	95
PAIN_M3_3	70	4.12	3.26	3.23	95	89
PAIN_M3_4	100	4.46	4.02	4.00	99	98
PAIN_M4_1	90	4.40	4.01	3.99	95	94
PAIN_M4_2	65	3.33	2.08	2.03	79	71
PAIN_M4_3	95	4.45	4.27	4.25	97	95
PAIN_F1_1	80	3.16	1.65	1.56	86	58
PAIN_F1_2	95	3.90	3.20	3.19	91	85
PAIN_F1_3	80	3.58	2.47	2.43	90	81
PAIN_F2_1	40	2.53	2.05	2.04	73	79
PAIN_F2_2	55	2.77	1.49	1.43	75	65
PAIN_F3_1	80	4.04	3.70	3.67	92	93
PAIN_F4_1	55	3.79	2.93	2.84	92	80
PAIN_F4_2	55	3.16	2.01	1.95	83	73
PAIN_F4_3	30	3.09	2.06	2.05	78	71
SATIETY_M1_1	30	1.18	– 1.61	– 1.81	33	20
SATIETY_M1_2	25	1.80	– 0.49	– 0.60	47	39
SATIETY_M2_1	35	1.73	– 0.79	– 0.89	43	36
SATIETY_M2_2	55	1.84	– 0.75	– 1.02	45	32
SATIETY_M3_1	45	1.59	– 1.28	– 1.44	43	25
SATIETY_M3_2	15	0.79	– 2.25	– 2.45	23	18
SATIETY_M4_1	50	1.84	– 0.56	– 0.71	45	38
SATIETY_M4_2	40	0.99	– 2.38	– 2.73	26	15
SATIETY_F1_1	35	1.22	– 1.57	– 1.82	34	19
SATIETY_F1_2	30	1.21	– 1.93	– 2.12	33	21
SATIETY_F2_1	20	0.78	– 2.30	– 2.47	21	15
SATIETY_F2_2	15	1.38	– 1.35	– 1.44	40	25
SATIETY_F3_1	50	1.68	– 0.73	– 0.91	42	35
SATIETY_F3_2	60	1.63	– 0.71	– 0.84	47	37
SATIETY_F3_3	70	2.66	1.27	1.16	64	59
SATIETY_F4_1	45	1.81	– 0.36	– 0.54	49	40
SATIETY_F4_2	50	1.79	– 0.48	– 0.58	47	41
BECKONING_M1_1	50	3.81	3.55	3.53	92	92
BECKONING_M1_2	50	4.03	4.01	4.00	92	94
BECKONING_M2_1	75	4.30	3.81	3.79	93	91
BECKONING_M2_2	65	4.14	3.95	3.95	92	93
BECKONING_M2_3	50	4.28	4.04	4.03	93	93
BECKONING_M2_4	50	4.24	3.98	3.96	93	92
BECKONING_M3_1	35	3.62	3.14	3.10	89	87
BECKONING_M3_2	40	3.81	3.66	3.63	91	93
BECKONING_M4_1	40	3.95	3.49	3.46	90	87
BECKONING_M4_2	45	3.49	3.16	3.13	85	85
BECKONING_F1_1	70	3.97	3.64	3.64	90	91
BECKONING_F1_2	50	3.81	3.53	3.52	91	91
BECKONING_F1_3	50	3.43	3.10	3.05	87	87
BECKONING_F2_1	30	2.47	1.91	1.89	77	75
BECKONING_F2_2	45	3.71	3.87	3.85	92	96
BECKONING_F3_1	50	3.17	2.68	2.67	83	84
BECKONING_F3_2	35	3.18	2.82	2.79	84	81
BECKONING_F4_1	55	3.26	2.71	2.70	82	85
BECKONING_F4_2	50	3.55	2.87	2.84	83	82
CLAPPING_M1_1	100	4.60	4.36	4.36	98	97
CLAPPING_M1_2	80	3.72	3.37	3.35	91	85
CLAPPING_M1_3	100	4.34	4.17	4.13	96	96
CLAPPING_M1_4	100	4.36	3.95	3.92	97	92
CLAPPING_M2_1	90	3.86	2.97	2.91	90	83
CLAPPING_M2_2	100	4.59	4.28	4.24	97	95
CLAPPING_M2_3	100	4.63	4.38	4.36	98	96
CLAPPING_M2_4	100	4.49	4.07	4.04	95	90
CLAPPING_M3_1	75	3.56	2.57	2.53	85	81
CLAPPING_M3_2	85	3.93	3.31	3.31	93	91
CLAPPING_M3_3	70	2.95	1.09	1.03	75	58
CLAPPING_M3_4	85	3.88	2.97	2.93	92	80
CLAPPING_M4_1	55	3.40	2.80	2.76	85	86
CLAPPING_M4_2	55	3.14	2.30	2.24	84	78
CLAPPING_M4_3	90	3.48	2.55	2.53	88	82
CLAPPING_M4_4	100	4.38	4.09	4.05	97	93
CLAPPING_F1_1	100	4.63	4.39	4.37	100	96
CLAPPING_F1_2	100	4.22	3.74	3.71	95	92
CLAPPING_F1_3	60	3.15	1.62	1.61	80	63
CLAPPING_F1_4	70	3.53	2.17	2.16	84	73
CLAPPING_F2_1	90	4.44	4.21	4.17	98	96
CLAPPING_F2_2	85	4.25	3.88	3.88	96	91
CLAPPING_F2_3	80	3.37	2.01	1.93	83	67
CLAPPING_F2_4	90	3.39	2.02	1.98	83	69
CLAPPING_F3_1	85	2.70	0.60	0.54	68	50
CLAPPING_F3_2	80	2.90	0.85	0.81	74	53
CLAPPING_F3_3	55	2.75	1.08	1.04	69	60
CLAPPING_F4_1	85	3.72	3.16	3.15	89	86
CLAPPING_F4_2	65	3.53	2.99	2.96	89	86
CLAPPING_F4_3	100	4.25	3.89	3.88	95	93
CLAPPING_F4_4	95	3.70	2.54	2.52	86	81
JUMPING_M1_1	85	3.52	2.06	1.98	81	73
JUMPING_M1_2	85	4.39	3.71	3.67	93	89
JUMPING_M1_3	95	4.34	3.89	3.89	94	93
JUMPING_M1_4	100	4.35	3.88	3.83	92	90
JUMPING_M3_1	65	3.08	2.71	2.67	80	82
JUMPING_M3_2	80	2.72	2.27	2.22	74	80
JUMPING_M4_1	75	2.73	1.82	1.79	71	71
JUMPING_M4_2	80	2.28	1.64	1.60	64	71
JUMPING_F1_1	65	3.14	3.09	3.02	81	88
JUMPING_F1_2	55	3.05	2.52	2.48	77	79
JUMPING_F1_3	90	3.23	3.15	3.15	84	91
JUMPING_F1_4	80	3.40	3.30	3.30	86	90
JUMPING_F2_1	50	3.27	2.88	2.84	86	85
JUMPING_F2_2	80	3.04	2.68	2.63	75	79
JUMPING_F2_3	100	4.35	4.19	4.18	98	96
JUMPING_F2_4	95	3.96	3.94	3.93	92	93
JUMPING_F3_1	90	3.59	3.24	3.22	86	87
JUMPING_F3_2	75	3.38	2.17	2.13	82	74
JUMPING_F3_3	60	3.71	3.38	3.37	85	88
JUMPING_F3_4	80	3.88	3.56	3.54	88	91
JUMPING_F4_1	75	2.79	2.10	2.09	70	76
JUMPING_F4_2	60	3.32	2.73	2.72	78	80
JUMPING_F4_3	85	2.90	2.42	2.37	71	76
LIFTING_M1_1	100	4.31	4.06	4.04	94	95
LIFTING_M1_2	75	4.22	3.82	3.80	91	93
LIFTING_M2_1	15	2.29	1.50	1.45	66	69
LIFTING_M2_2	55	3.55	2.89	2.87	85	82
LIFTING_M3_1	60	3.36	2.76	2.73	82	81
LIFTING_M3_2	35	2.89	2.36	2.34	73	77
LIFTING_M4_1	60	3.59	3.26	3.25	86	86
LIFTING_M4_2	65	3.82	3.09	3.06	86	84
LIFTING_M4_3	85	4.29	3.85	3.78	96	91
LIFTING_F1_1	85	4.04	3.82	3.78	87	90
LIFTING_F1_2	65	3.66	3.25	3.24	85	86
LIFTING_F1_3	80	3.85	3.59	3.55	85	91
LIFTING_F2_1	85	4.07	3.82	3.80	90	92
LIFTING_F2_2	10	2.34	2.14	2.13	70	81
LIFTING_F3_1	65	3.99	3.78	3.76	89	92
LIFTING_F3_2	65	3.40	2.60	2.55	84	79
LIFTING_F4_1	85	4.20	4.23	4.20	91	96
LIFTING_F4_2	65	3.98	3.68	3.65	86	88
LIFTING_F4_3	85	4.33	4.26	4.24	93	95
LIFTING_F4_4	60	3.59	2.97	2.90	84	85
RUNNING_M1_1	65	3.79	2.80	2.79	88	84
RUNNING_M1_2	90	3.75	2.93	2.91	88	83
RUNNING_M1_3	90	3.38	2.22	2.16	84	74
RUNNING_M2_1	95	3.89	3.39	3.34	95	91
RUNNING_M2_2	80	3.41	2.28	2.22	82	80
RUNNING_M2_3	100	3.89	3.04	3.02	92	81
RUNNING_M2_4	100	3.24	2.18	2.14	81	76
RUNNING_M3_1	100	4.23	3.80	3.75	95	92
RUNNING_M4_1	75	3.35	2.21	2.17	82	75
RUNNING_M4_2	70	2.72	1.26	1.20	73	59
RUNNING_M4_3	95	3.87	3.19	3.18	91	87
RUNNING_M4_4	90	3.75	2.77	2.74	90	81
RUNNING_F1_1	55	3.32	2.12	2.04	85	70
RUNNING_F1_2	55	2.37	0.90	0.85	69	58
RUNNING_F1_3	75	2.45	0.83	0.82	66	55
RUNNING_F1_4	70	3.03	1.86	1.83	79	67
RUNNING_F2_1	60	3.50	2.05	1.97	86	74
RUNNING_F2_2	85	3.52	2.35	2.33	84	74
RUNNING_F2_3	95	3.52	2.21	2.20	85	77
RUNNING_F2_4	90	4.03	3.61	3.60	94	92
RUNNING_F3_1	80	3.55	2.52	2.47	87	75
RUNNING_F3_2	100	4.61	4.34	4.29	97	96
RUNNING_F3_3	100	4.52	4.21	4.19	98	96
RUNNING_F3_4	100	4.24	3.70	3.68	92	88
RUNNING_F4_1	90	4.31	3.68	3.64	94	90
RUNNING_F4_2	85	3.17	1.66	1.62	77	67
TWIRLING_M1_1	45	2.10	0.60	0.52	59	54
TWIRLING_M1_2	65	2.83	1.62	1.55	75	66
TWIRLING_M1_3	60	2.89	2.16	2.12	77	73
TWIRLING_M2_1	80	3.65	3.31	3.28	91	92
TWIRLING_M3_1	55	3.93	3.60	3.56	90	89
TWIRLING_M3_2	65	3.71	3.39	3.35	88	89
TWIRLING_M3_3	90	3.22	2.60	2.58	81	80
TWIRLING_M4_1	50	2.89	1.96	1.95	76	71
TWIRLING_M4_2	85	2.99	1.96	1.94	79	71
TWIRLING_F1_1	50	3.03	1.35	1.34	76	64
TWIRLING_F1_2	70	3.94	3.55	3.49	90	90
TWIRLING_F1_3	90	3.66	2.91	2.86	87	84
TWIRLING_F1_4	95	3.72	2.98	2.95	86	85
TWIRLING_F2_1	50	1.81	– 0.46	– 0.58	51	41
TWIRLING_F2_2	60	2.47	0.63	0.54	62	56
TWIRLING_F3_1	60	3.83	3.27	3.25	89	87
TWIRLING_F3_2	55	3.16	1.28	1.25	73	66
TWIRLING_F4_1	45	3.38	2.90	2.86	83	84
TWIRLING_F4_2	80	3.30	2.43	2.39	79	76
WALKING_M1_1	100	4.24	3.97	3.97	97	95
WALKING_M1_2	100	3.95	3.71	3.69	93	92
WALKING_M1_3	90	3.76	3.48	3.47	91	91
WALKING_M1_4	65	3.37	3.35	3.34	86	91
WALKING_M2_1	100	4.19	3.85	3.84	96	95
WALKING_M2_2	95	3.06	2.24	2.21	78	78
WALKING_M2_3	80	3.61	3.26	3.19	90	89
WALKING_M2_4	75	2.78	3.05	3.04	76	91
WALKING_M3_1	100	3.10	1.84	1.82	77	67
WALKING_M3_2	100	4.10	3.80	3.80	93	93
WALKING_M3_3	90	3.57	2.43	2.40	90	78
WALKING_M3_4	100	4.28	4.12	4.10	96	95
WALKING_M4_1	95	3.75	3.55	3.55	92	95
WALKING_M4_2	90	2.69	1.79	1.70	75	70
WALKING_M4_3	80	3.41	3.22	3.20	85	89
WALKING_M4_4	95	3.73	3.48	3.43	91	88
WALKING_F1_1	100	4.28	4.11	4.09	95	94
WALKING_F1_2	100	4.29	4.11	4.09	95	94
WALKING_F1_3	80	2.81	2.29	2.27	74	78
WALKING_F1_4	55	2.74	2.67	2.64	75	84
WALKING_F2_1	95	3.56	3.13	3.12	88	87
WALKING_F2_2	85	3.62	3.29	3.29	90	92
WALKING_F2_3	65	2.04	2.45	2.45	60	83
WALKING_F2_4	100	4.27	4.13	4.10	96	97
WALKING_F3_1	95	3.59	3.38	3.34	89	90
WALKING_F3_2	95	4.27	4.11	4.11	96	98
WALKING_F3_3	100	4.32	4.16	4.14	93	94
WALKING_F3_4	100	4.03	3.78	3.78	96	95
WALKING_F4_1	95	4.04	3.84	3.83	94	95
WALKING_F4_2	100	4.02	3.81	3.80	89	92
WALKING_F4_3	100	4.15	3.94	3.91	96	96
WALKING_F4_4	90	3.84	3.28	3.27	90	90
WASHING_HANDS_M1_1	50	3.34	2.68	2.65	85	81
WASHING_HANDS_M1_2	55	3.86	3.37	3.35	90	88
WASHING_HANDS_M2_1	60	3.88	3.44	3.43	88	88
WASHING_HANDS_M2_2	60	3.20	2.31	2.23	82	76
WASHING_HANDS_M3_1	55	3.97	3.80	3.80	90	92
WASHING_HANDS_M3_2	55	3.10	2.13	2.10	76	75
WASHING_HANDS_M3_3	55	3.48	2.69	2.65	82	81
WASHING_HANDS_M4_1	30	3.08	2.55	2.52	83	83
WASHING_HANDS_M4_2	45	3.68	3.24	3.24	87	88
WASHING_HANDS_F1_1	45	4.15	3.42	3.40	95	88
WASHING_HANDS_F1_2	70	4.11	3.64	3.62	91	90
WASHING_HANDS_F1_3	65	4.20	3.54	3.49	90	86
WASHING_HANDS_F2_1	75	3.99	3.41	3.40	90	87
WASHING_HANDS_F2_2	60	3.77	3.26	3.25	87	88
WASHING_HANDS_F3_1	55	3.41	2.71	2.67	84	83
WASHING_HANDS_F3_2	65	3.67	3.28	3.26	87	87
WASHING_HANDS_F3_3	55	2.86	1.69	1.60	77	68
WASHING_HANDS_F4_1	65	3.82	3.04	2.98	85	85
WASHING_HANDS_F4_2	55	3.69	3.44	3.43	86	90
WAVING_M1_1	85	4.12	3.59	3.56	97	90
WAVING_M1_2	90	4.11	3.60	3.58	93	91
WAVING_M1_3	75	3.84	3.22	3.20	89	83
WAVING_M1_4	85	3.76	3.01	2.99	86	84
WAVING_M2_1	80	3.60	3.10	3.08	85	88
WAVING_M2_2	95	3.90	3.38	3.36	89	88
WAVING_M2_3	100	3.85	3.41	3.41	95	93
WAVING_M2_4	95	4.17	3.61	3.59	90	89
WAVING_M3_1	80	4.19	3.68	3.67	93	93
WAVING_M3_2	85	3.99	3.46	3.44	88	88
WAVING_M3_3	70	3.20	2.79	2.76	82	88
WAVING_M3_4	100	4.07	3.61	3.60	90	92
WAVING_M4_1	70	3.53	2.92	2.90	89	85
WAVING_M4_2	80	3.68	3.12	3.11	88	86
WAVING_M4_3	70	3.57	2.74	2.72	85	81
WAVING_M4_4	75	3.35	2.64	2.62	83	81
WAVING_F1_1	70	3.66	2.79	2.78	84	84
WAVING_F1_2	70	3.66	3.06	3.06	87	85
WAVING_F1_3	60	2.86	2.31	2.31	77	79
WAVING_F1_4	85	3.80	3.29	3.28	87	88
WAVING_F2_1	85	3.85	3.34	3.32	85	85
WAVING_F2_2	90	4.03	3.63	3.58	91	88
WAVING_F2_3	75	3.48	2.94	2.90	85	87
WAVING_F2_4	100	3.83	3.52	3.50	87	89
WAVING_F3_1	75	3.72	3.32	3.30	86	88
WAVING_F3_2	70	3.95	3.50	3.48	92	91
WAVING_F3_3	65	2.76	2.23	2.22	79	82
WAVING_F3_4	60	3.06	2.60	2.57	86	84
WAVING_F4_1	80	3.66	3.34	3.31	89	91
WAVING_F4_2	70	3.69	3.04	3.02	87	87
WAVING_F4_3	95	3.94	3.63	3.61	91	91
WAVING_F4_4	70	3.83	3.28	3.28	89	89

## Footnotes

1 It should be noted that these results may reflect the disproportionate presence of female raters in the sample. To account for gender differences in stimulus ratings, quality and accuracy scores from the validation data were analysed separately for female and male observers, as well as in the combined sample.
